# Optic Pathway Glioma in Children with Neurofibromatosis Type 1: A Multidisciplinary Entity, Posing Dilemmas in Diagnosis and Management Multidisciplinary Management of Optic Pathway Glioma in Children with Neurofibromatosis Type 1

**DOI:** 10.3389/fsurg.2022.886697

**Published:** 2022-05-03

**Authors:** Laura-Nanna Lohkamp, Patricia Parkin, Allan Puran, Ute Katharina Bartels, Eric Bouffet, Uri Tabori, James Thomas Rutka

**Affiliations:** ^1^ Division of Neurosurgery, Department of Surgery, The Hospital for Sick Children, Toronto, Ontario, Canada; ^2^ Division of Paediatric Medicine, Department of Paediatrics, The Hospital for Sick Children, Toronto, Ontario, Canada; ^3^ Division of Haematology/Oncology, Department of Paediatrics, The Hospital for Sick Children, Toronto, Ontario, Canada

**Keywords:** optic pathway glioma, neurofibromatosis type 1, diagnosis, management, multidisciplinary, neurofibromatosis clinic

## Abstract

**Introduction:**

Neurofibromatosis type 1 (NF1) has an incidence of 1 in 2,000 to 3,000 individuals and in 15% is associated with optic pathway glioma (OPG). Given the variability in clinical presentation and related morbidity, a multidisciplinary approach for diagnosis and management of children with NF1 and OPG is required, but often lacks coordination and regular information exchange. Herein we summarize our experience and describe the care pathways/network provided by a multidisciplinary team. The role of the distinct team members is elucidated as well as the care amendments made over time.

**Methods:**

We performed a retrospective single-center observational study, including children treated at our institution between 1990 and 2021. Inclusion criteria were clinical diagnosis of NF1, radiographic and/or histopathological diagnosis of OPG and age below 18 years. Patients being treated elsewhere were excluded from the study. Data was abstracted from each child’s health record using a standardized data collection form. Characteristics of children with NF1 and OPG were described using means (SD) and percentages. Outcomes were determined using Kaplan-Meier estimates.

**Results:**

From 1990 to 2021, 1,337 children were followed in our institution. Of those, 195 were diagnosed with OPG (14.6%), including 94 (48.21%) females and 101 (51.79%) males. Comprehensive data were available in 150 patients. The mean (SD) age at diagnosis was 5.31(4.08) years (range: 0.8–17.04 years). Sixty-two (41.3%) patients remained stable and did not undergo treatment, whereas 88 (58.7%) patients required at least one treatment. The mean (SD) duration of follow up was 8.14 (5.46) years (range: 0.1–25.9 years; median 6.8 years). Overall survival was of 23.6 years (±1.08), comprising 5 deaths. A dedicated NF clinic, including pediatricians and a nurse, provides regular follow up and plays a central role in the management of children with NF1, identifying those at risk of OPG, coordinating referrals to Neuroradiology and other specialists as indicated. All children are assessed annually by Ophthalmology. Comprehensive care was provided by a multidisciplinary team consisting of Dermatology, Genetics, Neuro-oncology, Neuroradiology, Neurosurgery, Ophthalmology and Pediatrics.

**Conclusions:**

The care of children with NF1 and OPG is optimized with a multidisciplinary team approach, coordinated by a central specialty clinic.

## Introduction

Neurofibromatosis type 1 (NF1) is an inherited neurocutaneous disorder occurring in about 1 in 2,000 to 1 in 3,000 individuals (1–4). Clinical features of NF1 are usually apparent in childhood, most often with multiple café au lait macules, and affect multiple organs, childhood development and neurocognitive status in a variable and age-dependant manner (5). As a familial tumor predisposition syndrome, it is characterized by the presence of multisystem tumors, which carry a risk of malignant transformation, and which are the major determinants of mortality in this population (6, 7). Optic Pathway Glioma (OPG) occur in 15%–20% of children with NF1 (8). They are found along the optic pathway and may involve one or both optic nerves, the chiasm, retro-chiasmatic structures including the hypothalamus, and the optic radiations (6). Although these tumors are typically low-grade gliomas, their behavior can be aggressive and therefore the clinical course may be highly variable, making treatment paradigms difficult (9, 10). Many children with NF1 and OPG remain asymptomatic. However, some children experience symptoms including vision loss and precocious puberty, highlighting the importance of early identification (11). Unfortunately, despite frequent imaging and ophthalmologic evaluations, some children experience progressive vision loss already before treatment, which makes their management even more challenging and emphasizes the need for optimized screening and follow-up protocols (12). Current management options include observation, surgery, chemotherapy, and radiation, all based on the current consensus to initiate treatment, when the tumor shows a substantial progression on MRI and/or becomes a threat to vision (13, 14). Given that OPG are not amenable to complete resection, the main treatment relies on chemotherapy (14). Other sequalae of OPG may include endocrinological deficits as well as primary or secondary neuropsychological limitations. Because of the varying features and clinical heterogeneity inherent to this condition, advocacy for a multidisciplinary approach to care and complex decision making has increased recently (8, 15, 16). However, the number of specialists involved may interfere with coordinated information exchange, referrals and follow up, thus posing dilemmas in the diagnosis and management of these patients. In 2007, the Children’s Tumor Foundation established a Neurofibromatosis Clinic Network in the United States, which aimed to standardize the level of care for individuals with NF and currently consists of 63 accredited clinics within the United States (17). Having established an NF specialty clinic in our institution in 1990, we seek to describe our experience, the evolution of care standards for children with NF1 related OPG over the last 30 years, the observed treatment paradigm changes as well as the characteristics of our patient cohort.

## Materials and Methods

### Study Design and Patient Cohort

We performed a single center, retrospective observational study at the Hospital for Sick Children (HSC) in Toronto, Canada, including children with NF1 and OPG diagnosed and treated between 1990 and 2021. Study subjects were identified from our NF1 registry, and their medical record number cross-referenced against information held in a second database, the Pediatric Brain Tumor Program registry. The study was approved by the local Research Ethics Board and conducted in accordance with the guidelines of the University of Toronto. Patient/parent consent was waived as de-identified data was abstracted from health records.

### Eligibility

Patients were included in the study when fulfilling the following criteria: (1) National Institutes of Health clinical diagnostic criteria (18) and/or *NF1* genetic testing (19), (2) confirmed OPG by neuro-imaging (MRI and/or CT), (3) age below 18 years and (4) received care at HSC. Patients, who did not meet these inclusion criteria or whose records were incomplete for more than 50% of the data points were excluded from the study.

### Data Extraction

Data was extracted from electronic patient records and included demographic, clinical, radiographic and follow up data at different time points over the entire course of each child’s care. Retrieved data aimed to describe the characteristics of children with NF1 and OPG, including presentation, clinical course (general and ophthalmologic examination), molecular genetic results, neuroimaging, management (observation, chemotherapy, surgery, radiotherapy) and outcome. De-identified data were collected using a standardized data collection form.

### Diagnosis and Treatment

A dedicated review of diagnostic and therapeutic procedures was performed, including the date and type of initial neuroimaging, the sequence of follow up examinations, duration of observation and treatment periods, treatment modalities and specific type (type of chemotherapy, surgery, or radiation) and treatment related complications. In case of surgical intervention, the patients’ charts were assessed for histopathological results. Post-treatment outcomes, indications for treatment changes or re-initiation were recorded and correlated with neuroimaging, ophthalmological, as well as clinical results. Molecular genetic testing is offered upon initial diagnosis and pursued according to parents’ preference. Neuropsychological assessments are provided either during neuro-oncological treatment or initiated via the NF clinic when learning difficulties or other neurodevelopmental deficits are identified during clinical follow up.

### Health Supervision

Children with confirmed or probable NF1 from infancy to 18 years are seen annually in the Pediatric NF Clinic following the health supervision guidelines from the American Academy of Pediatrics (20, 21). Children are also assessed annually in the Pediatric Ophthalmology Clinic. Children are followed by other members of the multidisciplinary team as required. In the early years of the clinic, universal screening and surveillance neuroimaging was recommended; however, currently we recommend neuroimaging as indicated according to medical or ophthalmologic concerns (22).

**Figure 1:**
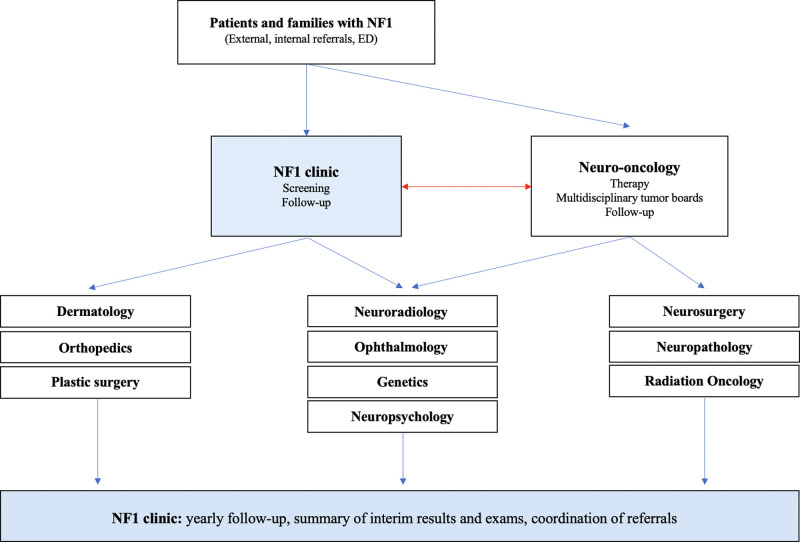
Schematic drawing illustrating the functional network of care for children with NF and OPG at the Hospital for Sick Children. The NF clinic plays a central role in connection, coordination, and integration of directed care for these complex patients.

**Figure 1** provides a schematic illustration of all specialties involved in the diagnosis and management of patients with NF1 and associated OPG.

### Outcome

The primary outcome was defined as overall survival (OS), describing the time period between diagnosis and last follow up. Follow up data of our patients, which were obtained from external institutions and available after the age of 18, were also included in the outcome analysis. Secondary outcomes were oncological status at the last visit, as well as presence of persisting visual and/or other sequalae.

### Statistical Analysis

Outcome was defined as OS and determined by Kaplan–Meier estimation (23). A confidence interval of 95% was applied and statistical significance was reached at a *p*-value equal or less than 0.05. All tests were performed using IBM SPSS Statistics Version 26.0 (IBM Corp. Released 2019. IBM SPSS Statistics for Windows, Version 26.0. Armonk, NY: IBM Corp, USA).

## Results

### General Demographics

**Figure 2:**
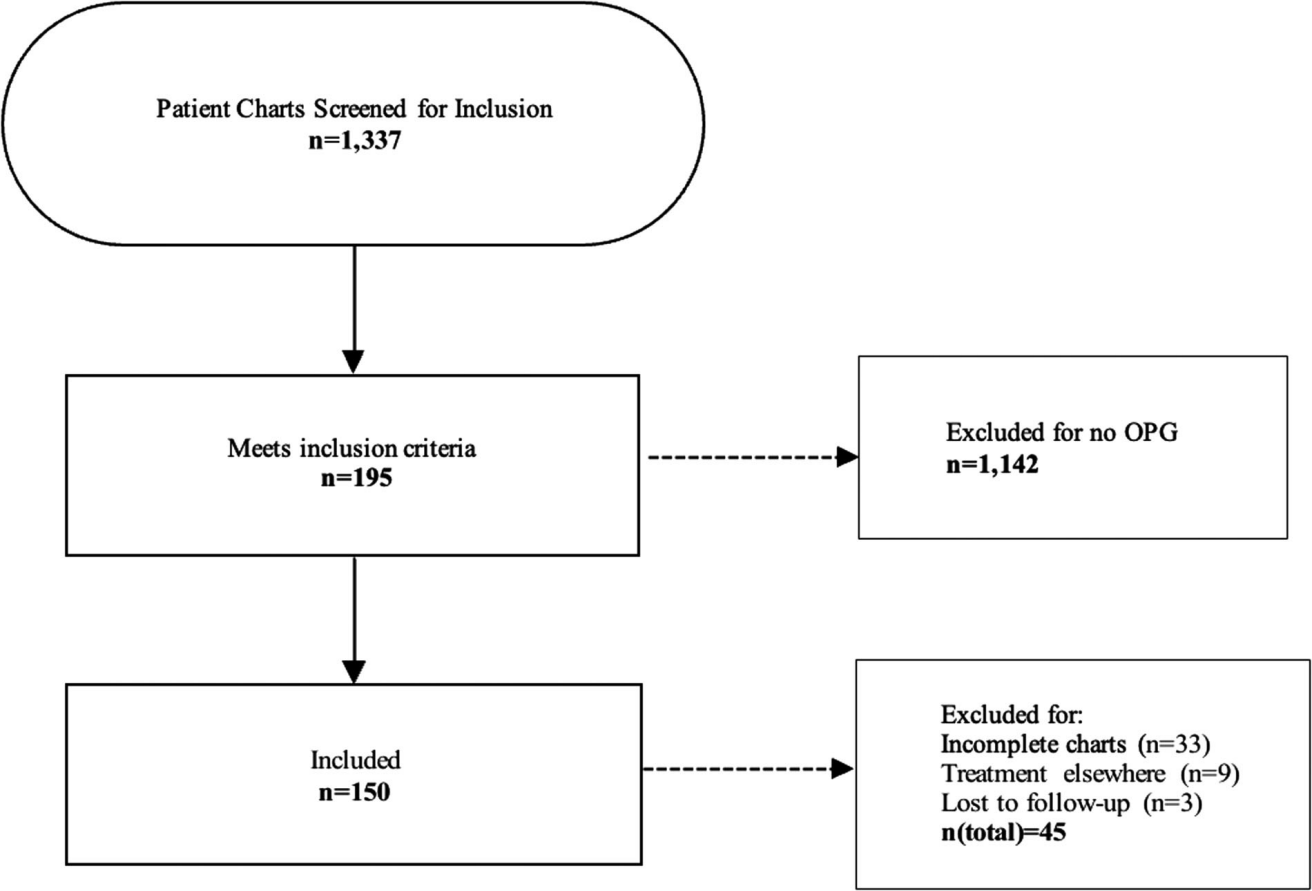
Flowchart illustrating the process of patient screening and the exclusion criteria applied. A final number of 150 patients diagnosed with NF and OPG was included in the study.

Between 1990 and 2021, 1,337 children with NF1 have been followed in the NF clinic and registered in the related database. Of those, 195 (14.6%) patients were radiographically diagnosed with OPG and met the inclusion criteria, including 94 (48.21%) females and 101 (51.79%) males. Forty-five patients were excluded from the study for the following reasons: 33 patients diagnosed between 1990 and 1998 had incomplete records, 9 patients were treated elsewhere, and 3 patients were lost to follow up (**Figure 2**). The mean (SD) age at diagnosis of the remaining 150 patients was 5.31(4.08) years (range: 0.8–17.04 years).

### Diagnosis

Diagnosis of OPG was obtained by neuroimaging, by CT in 19, by MRI in 131 patients. Thirty (20%) patients underwent neuroimaging as part of an NF screening process, whereas in 14 (9.3%) patients the finding of an OPG was incidental (obtained neuroimaging for other reasons) and resulted in further investigation of NF-related manifestations. Clinical presentation and symptoms leading to initial diagnostic imaging of the remaining 106 patients are listed in **Table 1**. Histopathological diagnosis was obtained in 53 patients: low grade astrocytoma not otherwise specified (NOS) (*n* = 20); pilocytic astrocytoma, (WHO grade I) (*n* = 27); pilomyxoid astrocytoma (WHO grade II) (*n* = 2); fibrillary astrocytoma (WHO grade II) (*n* = 1); and ganglioglioma (*n* = 1). Sixty-one patients were confirmed carrying mutations in the *NF1* gene by genetic testing.

**Table 1:** Clinical presentation of children leading to initial neuroimaging and diagnosis of OPG.

Clinical presentation	Number of Patients	%
Headaches	19	12.7
Vomiting	9	6
Visual symptoms	46	30.7
Visual signs	52	34.7
Cranial nerve palsy	4	2.7
Motor deficit	5	3.3
Seizures	4	2.7
Endocrinopathy	7	4.7
Macrocephaly	2	1.3
Developmental delay	22	14.7
Other	9	6

*The most common symptoms were related to vision. Some children presented with multiple symptoms.*

### Management

Being diagnosed with OPG, patients and their management were discussed in our multidisciplinary neuro-oncology rounds. Out of 150 patients, 62 (41.3%) remained stable and did not require any treatment, whereas 88 (58.7%) patients underwent at least one treatment throughout their course of disease. Of the thirty patients who were diagnosed with OPG via MRI screening, 10 (33%) patients required treatment during their clinical course. Four out of 14 (29%) patients whose OPG was an incidental finding were eventually submitted to treatment. Detailed treatment data were available in 150 children with OPG.

Initial management was observation in 84 (56%) patients, followed by chemotherapy (CTX) in 29 (19.3%) patients, and combined therapies in 21 (14%) patients. Upfront surgery alone was performed in 16 (10.7%) patients, including biopsies (*n* = 3), tumor debulking (*n* = 7), and gross total resection (GTR) (*n* = 6). A detailed list of initial management decisions is illustrated in **Table 2**.

**Table 2:** Treatment overview.

Initial management	Number of patients	%
Observation	84	56
CTX	29	19.3
Surgery (total)	16	10.7
Biopsy	3	2
Debulking	7	4.7
GTR	6	4
Combined therapies (total)	21	14
Biopsy + CTX	9	6
Debulking + CTX	10	6.7
GTR + RTX	2	1.3
Chemotherapy (total)	74	49.3
1 regimen	39	26
2 regimens	22	14.7
3 regimens	4	2.7
4 regimens	4	2.7
5 regimens	1	0.7
6 regimens	3	2
7 regimens	1	0.7
Radiotherapy	12	8
Primary treatment (+SX)	2	1.3
Secondary treatment	10	6.7
Surgery (secondary treatment)	8	5.3
Debulking	5	3.3
GTR	3	2
Hydrocephalus	30	20
Ventriculoperitoneal shunt	24	16
3rd Ventriculostomy	2	1.3
Ommaya reservoir insertion	1	0.7
Combined methods	3	2
Treatment summary/patient		
Observation	62	41.3
CTX	47	31.3
Debulking + CTX	17	11.3
Debulking + CTX + RTX	7	4.7
RTX	1	0.7
Debulking and RTX	3	2
GTR	8	5.3
Debulking	2	1.3
GTR + CTX	2	1.3
CTX + RTX	1	0.7

*CTX, chemotherapy; GTR, gross total resection; RTX, radiotherapy; SX, surgery.*

The main primary treatment was CTX, applied in 74 (49.3%) patients (see **Table 3** for chemotherapy protocols used as first-line treatment). Multiple regimens were required in 35 (23.3%) patients, due to tumor progression (*n* = 27) or CTX-related side effects (*n* = 8). Radiotherapy (RTX) was performed in a total of 12 (8%) patients, including 10 patients receiving radiation as a second-line treatment. Delayed tumor resection was required as secondary treatment in 8 (5.3%) patients. Clinical and radiographical signs of hydrocephalus were observed in 30 (20%) patients throughout their course of disease, being treated with ventriculoperitoneal (VP) shunts (*n* = 24), 3rd ventriculostomy (*n* = 2), Ommaya reservoir insertion (*n* = 1) and combined methods (*n* = 3).

**Table 3:** First chemotherapy regimen in children with OPG.

Chemotherapy protocol	Number of patients	%
Vincristine/Carboplatin (monthly)	15	20.3
Vincristine/Carboplatin 9952A (weekly)	17	23
Carboplatin/Etoposide/Vincristine	1	1.4
Vinblastine	25	33.8
Carboplatin only	9	12.2
Vincristine/Etoposide	1	1.4
TPCV^a^	1	1.4
Cyclophosphamide/Vincristine	1	1.4
Trametinib	2	2.7
Vinblastine/Bevacizumab	2	2.7

*A total of 74 children received chemotherapy. The initial regimen is listed and the number of receiving patients*.

^*a*^ *6-Thioguanine, Procarbazine, CCNU, and Vincristine*.

Over the decades a shift in treatment modalities was noted from upfront surgery in the 1990s to chemotherapy as first-line treatment in 2000 onwards. Also, the type of chemotherapy underwent changes over the years and shifted from Vincristine/Carboplatin to Vinblastine in the recent years. Radiation therapy has been applied in only 12 out of 150 patients, in 11 before and one after 2000. It has not been administered in any of the patients over the last 15 years. A detailed overview of therapy distributions is given in **Table 2** and these observations are more precisely described in the discussion section.

Neuropsychological testing was conducted in 100 (66.7%) patients, identifying 65 (65%) patients with learning disabilities and 29 (29%) with other mental health concerns (eg autism, attention deficit disorder).

Clinical follow up was provided on a 3-monthly basis during tumor treatment by the involved specialties (Neuro-oncology, Neuroradiology, Radiation Oncology, Ophthalmology, Neurosurgery) and for asymptomatic patients on a 6-monthly basis by Ophthalmology as well as on a yearly basis in the NF clinic.

### Clinical Outcome

The mean (SD) duration of follow up was 8.14 (5.46) years (range: 0.1–25.9 years; median 6.8 years). Five patients died during the study period; one after widespread dissemination of disease to the meninges and the brainstem after exhaustion of all therapy options; one from a radiotherapy induced secondary PNET which did not respond to treatment; two patients who were severely neurologically disabled from the effects of their tumor, progressed after completing one and three courses of chemotherapy, respectively, and no further treatment was attempted. The fifth patient, being severely compromised by multiple comorbidities died after a fatal tumor hemorrhage. For the remaining patients the status at last follow up was determined radiographically and/or clinically as stable in 132 (88%), in progression in 6 (4%), and in complete remission in 7 (4.7%) patients. Complete remission was defined as complete radiographic resolution of the OPG.

Tumor progression (ophthalmological: *n* = 9; radiographical: *n* = 18; both: *n* = 24; not documented: *n* = 10) after the first line treatment requiring further management was observed in 61 (40.7%) and included 22 (26.2%) out of 84 patients with initial observation only and 15 (51.7%) out 29 patients with initial chemotherapy. Decisions about second line treatments were made in our multidisciplinary tumor rounds and included start or change of CTX in 43 (28.6%), RTX in 10 (6.7%) and SX in 8 (5.3%) patients, respectively. A summary of all treatments administered per patient are indicated in **Table 2**.

**Figure 3:**
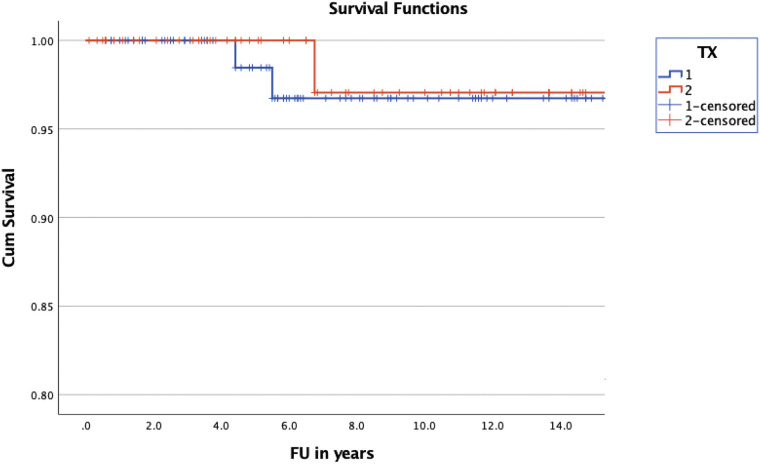
Kaplan-Meier curve illustrating the OS of children with NF1 and OPG. The difference of OS between treated (blue) and non-treated (red) children was statistically not significant (*p* = 0.455).

Overall survival was 25.23 years (±0.47) (mean; 95% CI of 24.3–26.16) in the treatment group and 24.49 years (±1.07) (mean; 95% CI of 18.4–22.57) in the observation group (**Figure 3**). The overall survival of the entire group was calculated with a mean of 23.6 years (±1.08) (95% CI of 21.48–25.72). Cumulative survival was 96.7 ± 2.3% in the treatment group and 97.1 ± 2.9% in the non-treatment group at 10 years of follow up. A log rank test comparing the OS of the treated and non-treated group did not confirm significance (*p* = 0.455).

Persisting visual deficits were noted in 29 (19.3%) patients at their last follow up, including 11 (7.3%) patients with unilateral and 5 (3.3%) patients with bilateral vision loss. A comprehensive list of all clinical features of NF1 patients with OPG is provided in **Table 4**.

**Table 4:** Clinical features of children with NF1 and OPG.

Clinical features	Number of patients	%
ADHD^a^	19	12.7
Audiological deficits	2	1.3
Endocrinopathy	29	19.3
Epilepsy	8	5.3
Learning disabilities	65	43.3
Neurological deficits	13	8.7
Other mental disorders	10	6.7
Second malignancy	2	1.3
Vasculopathy	10	6.7
Visual deficits	29	19.3

*Children with NF1 and OPG showed variable clinical features, which were documented at their last visit. Some of the children had acquired multiple diagnosis over their course of disease. Vasculopathies included two children with Moyamoya syndrome after radiation, three patients with a stroke after surgery, 3 patients with cerebral vasculopathies not otherwise specified, but without strokes, and 2 patients with renal artery stenosis.*

^*a*^*Attention deficit hyperactivity disorder*.

## Discussion

In this study we reviewed the clinical course of 150 children with NF1 and OPG, treated at HSC between 1990 and 2021. We analyzed the clinical outcome of both children who required treatment for OPG and those who underwent observation only. Despite many clinical features leading to an increased morbidity in this population, the estimated OS was 25.23 years (±0.467) in the treatment group and 24.487 years (±1.065) in the observation group, including 5 deaths. Treatment paradigm changes became visible in this retrospective study and will be discussed in the following. Furthermore, detailed analysis allowed us to shed light on the established treatment pathways at our institution, their evolution over time and integrative role within a multidisciplinary setting.

### Paradigm Shift in Treatment

Whereas surgery and radiation therapy were the initial mainstay therapies for OPG in 1990s, especially diagnostic biopsies followed by radiation and/or chemotherapy, the number of surgical interventions for OPG decreased significantly after 2000. This change occurred simultaneously with the introduction of our brain tumor program, which aimed to reduce treatment related side-effects in low-grade gliomas (LGG), and to establish standardized treatment approaches for children with NF1 and OPG according to their age and symptoms (14). Multiple studies were published around that time revisiting the optimal treatment strategy for these complex patients by investigating the role and risks of surgery. A report by *Nicolin et al.,* showed that aggressive debulking surgery had a negative impact on the neurocognitive outcomes of children with OPG. Patients treated with upfront debulking and chemotherapy displayed lower full and verbal scale IQ, than those treated with chemotherapy alone (24). Other studies confirmed the higher risk of neurological and/or endocrinological sequalae related to surgical therapy. *Valdueza et al*. reported their surgical experience in 20 patients with large hypothalamic/chiasmatic OPG (including 6 NF1 patients). Ten patients underwent debulking with resection of more than 50% of the tumor volume, 6 underwent partial resections, and 4 biopsies. The outcome was favorable in five patients with visual improvement following surgery. However, 4 patients had a visual decline, 1 patient developed a large cerebral infarction post-operatively and 4 patients suffered from endocrine complications (25). Similar observations were made by *Sawaruma et al*. They performed GTR in 7 patients with OPG, of which 5 patients experienced significant complications, which led the authors to the conclusion that the benefit of initial resection is questionable (26). Other studies, such as the report of *Ahn et al.* confirmed the surgery-related morbidity in a total of 33 patients, including 27 patients with GTR and 6 patients with debulking. Two patients in their study died of post-operative pulmonary embolism and diffuse cerebral infarction, 5 patients had a transient hemiparesis and 7 a visual deterioration (27). Furthermore, the benefit of aggressive surgery in OPG has been difficult to demonstrate with respect to tumor progression when compared to other upfront treatments (28). Different observations were made by *Liu et al.,* who observed in their study that residual tumor size was inversely associated with PFS in 165 patients with OPG and that adjuvant treatment could be avoided or deferred by maximized resection (29). Our cohort included 16 patients, who underwent upfront surgical treatment. Ten of them (GTR in 8 patients with isolated optic nerve glioma, debulking in 2 patients) did not require any further treatments. However, this number is by far too small for drawing comparative conclusions about the impact of tumor size reduction on PFS. With respect to surgical morbidity, we report 8 out of a total of 39 patients, who underwent either GTR or debulking, who suffered from significant neurological sequalae after surgery, including hemiparesis, focal deficits, and epilepsy, underlining the previously reported risk of surgical morbidity. Nevertheless, the role of surgery in NF 1 patients with OPG remains controversial and until recently there was no consensus for surgical management of these tumors except for unilateral optic nerve lesions associated with severe proptosis and/or complete unilateral blindness allowing for radical resection (14, 30). Surgical interventions for the treatment of related hydrocephalus or salvage surgeries follow a different argumentation and should be discussed individually for each patient.

Radiation therapy declined more drastically in its application for children with NF1 and OPG over the last 2 decades. It was considered an effective treatment for OPG in the past with high progression free survival rates (PFS) of up to 90% at 10 years (31–34). However, multiple studies have reported long-term side effects of radiation in patients with OPG, including endocrine abnormalities (33, 35), cerebrovascular disease (36–38), poor visual outcomes (31, 33, 39), secondary malignancies (40, 41), and neurocognitive deficits, particularly in young patients with developing brains (31, 42). *Capelli et al.* found that cerebrovascular complications after radiation were five times more frequent in children with OPG and NF1 than in those without NF1, and therefore calling for new treatment strategies in these patients (31). In a retrospective study *Ulrich et al.* analysed risk factors for the development of Moyamoya after radiation for primary brain tumors in 345 children. They observed that patients with NF1 carried a threefold increased risk of developing Moyamoya (HR = 3.07, 95% CI, 0.90–10.46, *p* = 0.07) besides an earlier onset of Moyamoya compared to non-NF1 patients (median of 38 vs 55 months) (38). Because patients with NF1 have a greater risk of neoplasms and cerebrovascular disease at baseline, these adverse outcomes are of particular concern in the NF1 population. Hence radiation therapy has become a therapy of last resort, reserved for adolescent patients and those with no remaining chemotherapeutic or targeted treatment options (43). In our institution, a total of 12 children with NF1 and OPG underwent radiation therapy over the last 31 years, 11 of them irradiated before 2000, when our brain tumor program was initiated. Two of those patients developed Moyamoya post radiation and required a cerebral revascularization procedure. However, there are newer methods of radiation, such as proton beam radiation therapy and stereotactic radiosurgery. These have shown positive short-term results in NF1 patients with OPG and may be potential (salvage) treatment options in the future, depending on their currently investigated long-term outcomes (43–45).

Beyond the shift of primary treatment towards chemotherapy, we observed additional changes within the first line chemotherapy regimens administered in children with NF1 and OPG. In line with the former recommendations, the regimen we mainly used in the 1990s was a combination of Vincristine and Carboplatin (46). Other regimens followed until a cutoff was noted in 2007 with predominant prescription of either Vinblastine alone or recently also Vinblastine in combination with Bevacizumab. This change was a response to the common adverse effects of other chemotherapies, including neurotoxicity (vincristine), hypersensitivity (carboplatin) (47, 48), ototoxicity (49) (cisplatin), infertility, and other long-term toxicities with alkylating agents (procarbazine and lomustine) or increased risk of secondary malignancies with etoposide (50). *Lassaletta et al.* reported a multicenter study, which investigated the comparative effectiveness and safety of a single-agent first-line chemotherapy with Vinblastine in pediatric low-grade glioma (LGG), including 13 patients with NF1. They showed that Vinblastine was well tolerated by all children while representing a less toxic treatment with similar efficacy, in terms of OS, PFS, and visual outcomes, as other regimens. Interestingly, patients with NF1 had a significantly better PFS (85.1%; 95% CI, 68.0%–100%) when compared to patients without NF1 (42.0%; 95% CI, 29.1%–60.7%; *P* = .012), identifying Vinblastine as an optimal agent for children with NF1 and OPG (51). Most recently, combinations of Vinblastine and Bevacizumab have emerged as promising regimens for OPG (52, 53). Bevacizumab is an anti-vascular endothelial growth factor (VEGF) monoclonal antibody, which reduces neovascularization as well as tumor growth by targeting VEGF. It has been previously used in other childhood malignancies and demonstrated good tolerability (54, 55). Current applications in patients with NF1 and OPG are refractory patients until further studies elucidate its comprehensive effect on clinical outcomes (52).

### Screening (MRI Algorithm)

Routine MRI screening for OPG in children with NF1 has been a topic of controversy over the last decade. Despite the high incidence of OPG in the NF1 population, brain MRI is not recommended as a screening tool, as treatment is not indicated in the absence of visual symptoms or proptosis (14). Serial visual assessment is currently considered the standard screening tool for OPG, including yearly visual assessment up to the age of 8 years and every other year until the age of 18 years (22). However, ophthalmological exams in the youngest children, which are also at highest risk for developing OPG can be challenging and requires relevant expertise (14, 56). Furthermore, in the context of a tumor that involves the optic pathway, loss of visual acuity may be a late occurrence and efforts to detect a threat to vision should ideally be identified earlier. Prevention of visual deficits is one of the major goals in following NF1 patients with OPG and awaiting the ophthalmologist to detect a visual decline as evidence for tumor progression may be a justified critique. However, early detection of an asymptomatic OPG has not been proven to reduce the incidence of visual loss, nor does an initial normal MRI exclude the development of a subsequent OPG (57, 58). But there are more factors to consider. Similar to the observation in our cohort, a significant number of children with NF1 and OPG remain asymptomatic and do not require any treatment throughout their entire clinical course (16). A study by *Listernick et al.* in 1989 demonstrated that, if all children with NF1 undergo screening neuroimaging, 15% of them will have radiographic evidence of OPG with only half of these children developing symptoms or signs related to the OPG (59). We followed 1337 children with NF in our specialty clinic, of which 195 (14.6%) were diagnosed with OPG. Unfortunately, complete data were only available for 150 patients (11.2%), including 88 treated and 62 asymptomatic patients, who never required any intervention. This corresponds roughly to the results of *Listernick et al.* and underlines that only a small group of patients would benefit from serial MRI screening – in our study 88 (6.6%) patients out of 1,337. Given that MR Imaging is an expensive, time-consuming exam, which requires the use of anesthetics in young patients (mostly until the age of 5–6 years), the costs, risk of general anesthesia and recourse control outweigh the benefit of serial screening MR Imaging in NF1 patients. Additionally, few studies reported spontaneous regression of low-grade OPG (60, 61). Taking these aspects into consideration, the recommendation of primary ophthalmological and clinical screening for OPG was implemented as diagnostic standard in our institution and is coordinated by our NF clinic.

### Role of NF Clinic

Multiple factors have led in the past to the establishment and integration of specialized pediatric NF clinics in major therapy centers (15–17, 62, 63). First, the complexity of the disease and its requirements for comprehensive diagnosis and follow up are important and exceed the competences and capacities of single specialists, such as oncologists, neuroradiologists, neurosurgeons, or ophthalmologists (15). Children with NF may present simultaneously with multiple clinical features and are constantly at risk of developing malignancies (64, 65), which may have a significant impact on their life expectancy (17). This results in a high-intensity demand of care, coordination of multiple specialties and integration of continuous exam results. Second, symptoms such as rapid expansion of existing tumors, chronic pain, and neurological deficits require immediate medical attention, dedicated examination, and treatment (66). Age-specific monitoring of symptoms and education of patients and their parents are important in managing NF1 patients and should be provided by a pediatrician specialized in NF (15, 67). Our NF1 clinic was established in 1990, consists of 3 pediatricians and one nurse, having provided care for 1,337 children with NF1 to date. Its role is threefold and includes diagnosis of NF1, regular follow up and screening for new malignancies. Patients undergo a comprehensive diagnostic work-up at presentation, including genetic testing if desired by the parents/patient, followed by yearly follow up visits. Referrals to other specialties are made upon clinical needs and diagnosis, except for ophthalmology, which is part of a regulated follow up schedule. According to the recommendations of the NF1 Optic Pathway Glioma Task Force the children undergo yearly visual assessment until the age of 8 years and assessment every other year until the age of 18 years (22). Having established this central specialty clinic for children with NF1, the coordination of the patient’s care as well as their maintenance of follow-up has improved significantly. Further studies will aim to objectify the impact on care via a dedicated NF1 clinic by reviewing data on diagnosis of malignancies, referral pathways and adherence to follow up. Lastly, we could also observe a significant advantage of the NF1 clinic with respect to reviewing and updating other specialty exam results and with respect to collecting outcome data in a comprehensive manner for all NF associated clinical features. This sort of surveillance may contribute positively to care optimization and institutional quality assessment. Other considerations of NF specialty clinics are their guidance for facilitating transition and continuation of care in adult NF patients. Adult counterpart clinics may provide purposeful follow up while maintaining the same level of expertise and quality of care and therefore represent an equally important institution after transition from pediatrics to adulthood (68).

### Limitations

There are several limitations to the study. First, a relevant number of patients had to be excluded from the study due to missing data, which might compromise the significance of our results. Second, we did not report detailed neuro-ophthalmologic results of visual outcome. Although it is one of the most important determinants of OPG management and outcome, we focused on OS and overall clinical status at patients’ discharge to adult care. The reason we did not report visual outcome in detail was to avoid redundance as it was previously described by Nicolin et al (24). However, the overall visual outcome was considered when evaluating the clinical status at last follow up. Patients, who were categorized it as clinically stable, also had a stable exam in their last ophthalmological follow up. The number of patients with persisting visual deficits are summarized in **Table 4**. Lastly, further limitations of our study are reflected by missing subgroup analysis regarding the different treatment constellations and their impact on OS. Acknowledging the highly variable and to some extent small patient numbers per subgroup (compare treatment summary/patient in **Table 2**), we did not consider its results as sufficiently robust for generalizability to all pediatric populations with NF1 and OPG.

## Conclusion

This report confirms the complexity of the natural history of OPG, its requirement for multidisciplinary management and the beneficial role of a centrally integrated NF1 clinic. Treatment algorithms experienced several changes over the last decades and will be subject to constant optimization. Specialized NF clinics represent a substantial element in the care network for children with NF1 and OPG, supporting coordination of several multidisciplinary clinics, offering specialized and comprehensive care besides serving epidemiological aspects by assembling data of these patients in a centralized manner. Having this integrative role, dedicated NF1 clinics should become a regular component in the multidisciplinary management of patients with NF and OPG.

## Data Availability

The raw data supporting the conclusions of this article will be made available by the authors upon request, without undue reservation.

## Nomenclature

CT: Computed Tomography
CTX: Chemotherapy
HSC: Hospital for Sick Children
MRI: Magnet Resonance Imaging
NF1: Neurofibromatosis (type 1)
OPG: Optic Pathway Glioma
OS: Overall Survival
PFS: Progression Free Survival
RTX: Radiation Therapy
SD: Standard Deviation
SX: Surgery
VP shunt: Ventriculoperitoneal Shunt

## References

[1] Kissil JL Blakeley JO Ferner RE Huson SM Kalamarides M Mautner VF What’s new in neurofibromatosis? Proceedings from the 2009 NF Conference: new frontiers. Am J Med Genet A. 152a(2):269–83. 10.1002/ajmg.a.33189 20082461 PMC2818482

[2] Evans DG Howard E Giblin C Clancy T Spencer H Huson SM Birth incidence and prevalence of tumor-prone syndromes: estimates from a UK family genetic register service. Am J Med Genet A. (2010) 152a(2):327–32. 10.1002/ajmg.a.33139 20082463

[3] Uusitalo E Leppävirta J Koffert A Suominen S Vahtera J Vahlberg T Incidence and mortality of neurofibromatosis: a total population study in Finland. J Invest Dermatol. (2015) 135(3):904–6. 10.1038/jid.2014.465 25354145

[4] Lammert M Friedman JM Kluwe L Mautner VF. Prevalence of neurofibromatosis 1 in German children at elementary school enrollment. Arch Dermatol. (2005) 141(1):71–4. 10.1001/archderm.141.1.71 15655144

[5] Evans DGR Salvador H Chang VY Erez A Voss SD Schneider KW Cancer and central nervous system tumor surveillance in pediatric neurofibromatosis 1. Clin Cancer Res. (2017) 23(12):e46–e53. 10.1158/1078-0432.Ccr-17-0589 28620004

[6] Shofty B Ben Sira L Constantini S. Neurofibromatosis 1-associated optic pathway gliomas. Childs Nerv Syst. (2020) 36(10):2351–61. 10.1007/s00381-020-04697-1 32524182

[7] Tadini G Milani D Menni F Pezzani L Sabatini C Esposito S. Is it time to change the neurofibromatosis 1 diagnostic criteria? Eur J Intern Med. (2014) 25(6):506–10. 10.1016/j.ejim.2014.04.004 24784952

[8] Hirbe AC Gutmann DH. Neurofibromatosis type 1: a multidisciplinary approach to care. Lancet Neurol. (2014) 13(8):834–43. 10.1016/s1474-4422(14)70063-8 25030515

[9] Binning MJ Liu JK Kestle JR Brockmeyer DL Walker ML. Optic pathway gliomas: a review. Neurosurg Focus. (2007) 23(5):E2. 10.3171/foc-07/11/e2 18004964

[10] Chong AL Pole JD Scheinemann K Hukin J Tabori U Huang A Optic pathway gliomas in adolescence–time to challenge treatment choices? Neuro-oncology. (2013) 15(3):391–400. 10.1093/neuonc/nos312 23295772 PMC3578487

[11] Karaconji T Whist E Jamieson RV Flaherty MP Grigg JRB. Neurofibromatosis type 1: review and update on emerging therapies. Asia Pac J Ophthalmol (Phila). (2019) 8(1):62–72. 10.22608/apo.2018182 30387339

[12] de Blank PMK Fisher MJ Liu GT Gutmann DH Listernick R Ferner RE Optic pathway gliomas in neurofibromatosis type 1: an update: surveillance, treatment indications, and biomarkers of vision. J Neuroophthalmol. (2017) 37 Suppl 1(Suppl 1):S23–s32. 10.1097/wno.0000000000000550 28806346 PMC7410089

[13] Fisher MJ Loguidice M Gutmann DH Listernick R Ferner RE Ullrich NJ Visual outcomes in children with neurofibromatosis type 1-associated optic pathway glioma following chemotherapy: a multicenter retrospective analysis. Neuro-oncology. (2012) 14(6):790–7. 10.1093/neuonc/nos076 22474213 PMC3367846

[14] Fried I Tabori U Tihan T Reginald A Bouffet E. Optic pathway gliomas: a review. CNS Oncol. (2013) 2(2):143–59. 10.2217/cns.12.47 25057976 PMC6169473

[15] Nishida Y Ikuta K Natsume A Ishihara N Morikawa M Kidokoro H Establishment of in-hospital clinical network for patients with neurofibromatosis type 1 in Nagoya University Hospital. Sci Rep. (2021) 11(1):11933. 10.1038/s41598-021-91345-6 34099792 PMC8184989

[16] Kokkinou E Roka K Alexopoulos A Tsina E Nikas I Krallis P Development of a multidisciplinary clinic of neurofibromatosis type 1 and other neurocutaneous disorders in Greece. A 3-year experience. Postgrad Med. (2019) 131(7):445–52. 10.1080/00325481.2019.1659708 31443616

[17] Merker VL Dai A Radtke HB Knight P Jordan JT Plotkin SR. Increasing access to specialty care for rare diseases: a case study using a foundation sponsored clinic network for patients with neurofibromatosis 1, neurofibromatosis 2, and schwannomatosis. BMC Health Serv Res. (2018) 18(1):668. 10.1186/s12913-018-3471-5 30157837 PMC6114484

[18] National Institutes of Health. National institutes of health consensus development conference statement: neurofibromatosis. Bethesda, Md., USA, July 13–15, 1987. Neurofibromatosis. (1988) 1(3):172–8. 3152465

[19] Legius E Messiaen L Wolkenstein P Pancza P Avery RA Berman Y Revised diagnostic criteria for neurofibromatosis type 1 and Legius syndrome: an international consensus recommendation. Genet Med. (2021) 23(8):1506–13. 10.1038/s41436-021-01170-5 34012067 PMC8354850

[20] Hersh JH. Health supervision for children with neurofibromatosis. Pediatrics. (2008) 121(3):633–42. 10.1542/peds.2007-3364 18310216

[21] Miller DT Freedenberg D Schorry E Ullrich NJ Viskochil D Korf BR. Health supervision for children with neurofibromatosis type 1. Pediatrics. (2019) 143(5): e20190660. 10.1542/peds.2019-0660 31010905

[22] Listernick R Louis DN Packer RJ Gutmann DH. Optic pathway gliomas in children with neurofibromatosis 1: consensus statement from the NF1 Optic Pathway Glioma Task Force. Ann Neurol. (1997) 41(2):143–9. 10.1002/ana.410410204 9029062

[23] Kaplan EL. MPNefi, 1958;53:457–481. oJASA.

[24] Nicolin G Parkin P Mabbott D Hargrave D Bartels U Tabori U Natural history and outcome of optic pathway gliomas in children. Pediatr Blood Cancer. (2009) 53(7):1231–7. 10.1002/pbc.22198 19621457

[25] Valdueza JM Lohmann F Dammann O Hagel C Eckert B Freckmann N. Analysis of 20 primarily surgically treated chiasmatic/hypothalamic pilocytic astrocytomas. Acta Neurochir. (1994) 126(1):44–50. 10.1007/bf01476493 8154322

[26] Sawamura Y Kamada K Kamoshima Y Yamaguchi S Tajima T Tsubaki J Role of surgery for optic pathway/hypothalamic astrocytomas in children. Neuro-oncology. (2008) 10(5):725–33. 10.1215/15228517-2008-033 18612049 PMC2666249

[27] Ahn Y Cho BK Kim SK Chung YN Lee CS Kim IH Optic pathway glioma: outcome and prognostic factors in a surgical series. Childs Nerv Syst. (2006) 22(9):1136–42. 10.1007/s00381-006-0086-7 16628460

[28] Steinbok P Hentschel S Almqvist P Cochrane DD Poskitt K. Management of optic chiasmatic/hypothalamic astrocytomas in children. Can J Neurol Sci. (2002) 29(2):132–8. 12035834

[29] Liu ZM Liao CH An X Zhou WT Ma ZY Liu W The role of imaging features and resection status in the survival outcome of sporadic optic pathway glioma children receiving different adjuvant treatments. Neurosurg Rev. (2022). 10.1007/s10143-022-01743-1 35106677

[30] Zeid JL Charrow J Sandu M Goldman S Listernick R. Orbital optic nerve gliomas in children with neurofibromatosis type 1. J AAPOS. (2006) 10(6):534–9. 10.1016/j.jaapos.2006.03.014 17189147

[31] Cappelli C Grill J Raquin M Pierre-Kahn A Lellouch-Tubiana A Terrier-Lacombe MJ Long-term follow up of 69 patients treated for optic pathway tumours before the chemotherapy era. Arch Dis Child. (1998) 79(4):334–8. 10.1136/adc.79.4.334 9875044 PMC1717725

[32] Grabenbauer GG Schuchardt U Buchfelder M Rodel CM Gusek G Marx M Radiation therapy of optico-hypothalamic gliomas (OHG)–radiographic response, vision and late toxicity. Radiother Oncol. (2000) 54(3):239–45. 10.1016/s0167-8140(00)00149-3 10738082

[33] Horwich A Bloom HJ. Optic gliomas: radiation therapy and prognosis. Int J Radiat Oncol Biol Phys. (1985) 11(6):1067–79. 10.1016/0360-3016(85)90052-5 3997589

[34] Jenkin D Angyalfi S Becker L Berry M Buncic R Chan H Optic glioma in children: surveillance, resection, or irradiation? Int J Radiat Oncol Biol Phys. (1993) 25(2):215–25. 10.1016/0360-3016(93)90342-s 8420869

[35] Pierce SM Barnes PD Loeffler JS McGinn C Tarbell NJ. Definitive radiation therapy in the management of symptomatic patients with optic glioma. Survival and long-term effects. Cancer. (1990) 65(1):45–52. 10.1002/1097-0142(19900101)65:1<45::aid-cncr2820650111>3.0.co;2-z 2104571

[36] Grill J Couanet D Cappelli C Habrand JL Rodriguez D Sainte-Rose C Radiation-induced cerebral vasculopathy in children with neurofibromatosis and optic pathway glioma. Ann Neurol. (1999) 45(3):393–6. 10.1002/1531-8249(199903)45:3<393::aid-ana17>3.0.co;2-b 10072056

[37] Kestle JR Hoffman HJ Mock AR. Moyamoya phenomenon after radiation for optic glioma. J Neurosurg. (1993) 79(1):32–5. 10.3171/jns.1993.79.1.0032 8315466

[38] Ullrich NJ Robertson R Kinnamon DD Scott RM Kieran MW Turner CD Moyamoya following cranial irradiation for primary brain tumors in children. Neurology. (2007) 68(12):932–8. 10.1212/01.wnl.0000257095.33125.48 17372129

[39] Bataini JP Delanian S Ponvert D. Chiasmal gliomas: results of irradiation management in 57 patients and review of literature. Int J Radiat Oncol Biol Phys. (1991) 21(3):615–23. 10.1016/0360-3016(91)90678-w 1907959

[40] Sharif S Ferner R Birch JM Gillespie JE Gattamaneni HR Baser ME Second primary tumors in neurofibromatosis 1 patients treated for optic glioma: substantial risks after radiotherapy. J Clin Oncol. (2006) 24(16):2570–5. 10.1200/jco.2005.03.8349 16735710

[41] Tsang DS Murphy ES Merchant TE. Radiation therapy for optic pathway and hypothalamic low-grade gliomas in children. Int J Radiat Oncol Biol Phys. (2017) 99(3):642–51. 10.1016/j.ijrobp.2017.07.023 29280458

[42] Lacaze E Kieffer V Streri A Lorenzi C Gentaz E Habrand JL Neuropsychological outcome in children with optic pathway tumours when first-line treatment is chemotherapy. Br J Cancer. (2003) 89(11):2038–44. 10.1038/sj.bjc.6601410 14647135 PMC2376861

[43] Farazdaghi MK Katowitz WR Avery RA. Current treatment of optic nerve gliomas. Curr Opin Ophthalmol. (2019) 30(5):356–63. 10.1097/icu.0000000000000587 31246635 PMC7410088

[44] El-Shehaby AM Reda WA Abdel Karim KM Emad Eldin RM Nabeel AM. Single-session Gamma Knife radiosurgery for optic pathway/hypothalamic gliomas. J Neurosurg. (2016) 125(Suppl 1):50–57. 10.3171/2016.8.Gks161432 27903182

[45] Fuss M Hug EB Schaefer RA Nevinny-Stickel M Miller DW Slater JM Proton radiation therapy (PRT) for pediatric optic pathway gliomas: comparison with 3D planned conventional photons and a standard photon technique. Int J Radiat Oncol Biol Phys. (1999) 45(5):1117–26. 10.1016/s0360-3016(99)00337-5 10613303

[46] Ater JL Zhou T Holmes E Mazewski CM Booth TN Freyer DR Randomized study of two chemotherapy regimens for treatment of low-grade glioma in young children: a report from the Children’s Oncology Group. J Clin Oncol. (2012) 30(21):2641–7. 10.1200/jco.2011.36.6054 22665535 PMC3413276

[47] Lafay-Cousin L Sung L Carret AS Hukin J Wilson B Johnston DL Carboplatin hypersensitivity reaction in pediatric patients with low-grade glioma: a Canadian Pediatric Brain Tumor Consortium experience. Cancer. (2008) 112(4):892–9. 10.1002/cncr.23249 18098210

[48] Yu DY Dahl GV Shames RS Fisher PG. Weekly dosing of carboplatin increases risk of allergy in children. J Pediatr Hematol Oncol. (2001) 23(6):349–52. 10.1097/00043426-200108000-00005 11563768

[49] Orts Alborch M García Callejo J Morant Ventura A Ferrer Baixauli F Esparcia Navarro M Marco Algarra J. Clinical study on the ototoxicity of cisplatin with distortion products. Acta Otorrinolaringol Esp. (2000) 51(6):469–77. 11142781

[50] Nageswara Rao AA Packer RJ. Advances in the management of low-grade gliomas. Curr Oncol Rep. (2014) 16(8):398. 10.1007/s11912-014-0398-9 24925153

[51] Lassaletta A Scheinemann K Zelcer SM Hukin J Wilson BA Jabado N Phase II weekly vinblastine for chemotherapy-naïve children with progressive low-grade glioma: a canadian pediatric brain tumor consortium study. J Clin Oncol. (2016) 34(29):3537–43. 10.1200/jco.2016.68.1585 27573663

[52] Avery RA Hwang EI Jakacki RI Packer RJ. Marked recovery of vision in children with optic pathway gliomas treated with bevacizumab. JAMA Ophthalmol. (2014) 132(1):111–4. 10.1001/jamaophthalmol.2013.5819 24232489

[53] Zhukova N Rajagopal R Lam A Coleman L Shipman P Walwyn T Use of bevacizumab as a single agent or in adjunct with traditional chemotherapy regimens in children with unresectable or progressive low-grade glioma. Cancer Med. (2019) 8(1):40–50. 10.1002/cam4.1799 30569607 PMC6346232

[54] Roux C Revon-Rivière G Gentet JC Verschuur A Scavarda D Saultier P Metronomic maintenance with weekly vinblastine after induction with bevacizumab-irinotecan in children with low-grade glioma prevents early relapse. J Pediatr Hematol Oncol. (2021) 43(5):e630–34. 10.1097/mph.0000000000002002 33235152

[55] Glade Bender JL Adamson PC Reid JM Xu L Baruchel S Shaked Y Phase I trial and pharmacokinetic study of bevacizumab in pediatric patients with refractory solid tumors: a Children’s Oncology Group Study. J Clin Oncol. (2008) 26(3):399–405. 10.1200/jco.2007.11.9230 18202416

[56] Avery RA Hwang EI Ishikawa H Acosta MT Hutcheson KA Santos D Handheld optical coherence tomography during sedation in young children with optic pathway gliomas. JAMA Ophthalmol. (2014) 132(3):265–71. 10.1001/jamaophthalmol.2013.7649 24435762 PMC4445404

[57] Gutmann DH Aylsworth A Carey JC Korf B Marks J Pyeritz RE The diagnostic evaluation and multidisciplinary management of neurofibromatosis 1 and neurofibromatosis 2. JAMA. (1997) 278(1):51–7. 9207339

[58] Cassiman C Legius E Spileers W Casteels I. Ophthalmological assessment of children with neurofibromatosis type 1. Eur J Pediatr. (2013) 172(10): 1327–33. 10.1007/s00431-013-2035-2 23708214

[59] Listernick R Charrow J Greenwald MJ Esterly NB. Optic gliomas in children with neurofibromatosis type 1. J Pediatr. (1989) 114(5):788–92. 10.1016/s0022-3476(89)80137-4 2497236

[60] Parsa CF Hoyt CS Lesser RL Weinstein JM Strother CM Muci-Mendoza R Spontaneous regression of optic gliomas: thirteen cases documented by serial neuroimaging. Arch Ophthalmol. (2001) 119(4):516–29. 10.1001/archopht.119.4.516 11296017

[61] Piccirilli M Lenzi J Delfinis C Trasimeni G Salvati M Raco A. Spontaneous regression of optic pathways gliomas in three patients with neurofibromatosis type I and critical review of the literature. Childs Nerv Syst. (2006) 22(10):1332–7. 10.1007/s00381-006-0061-3 16639629

[62] McKeever K Shepherd CW Crawford H Morrison PJ. An epidemiological, clinical and genetic survey of neurofibromatosis type 1 in children under sixteen years of age. Ulster Med J. (2008) 77(3):160–3. 18956796 PMC2604471

[63] Rosenfeld A Listernick R Charrow J Goldman S. Neurofibromatosis type 1 and high-grade tumors of the central nervous system. Childs Nerv Syst. (2010) 26(5):663–7. 10.1007/s00381-009-1024-2 19937438

[64] Yohay K. Neurofibromatosis type 1 and associated malignancies. Curr Neurol Neurosci Rep. (2009) 9(3):247–53. 10.1007/s11910-009-0036-3 19348714

[65] Seminog OO Goldacre MJ. Risk of benign tumours of nervous system, and of malignant neoplasms, in people with neurofibromatosis: population-based record-linkage study. Br J Cancer. (2013) 108(1):193–8. 10.1038/bjc.2012.535 23257896 PMC3553528

[66] Créange A Zeller J Rostaing-Rigattieri S Brugières P Degos JD Revuz J Neurological complications of neurofibromatosis type 1 in adulthood. Brain. (1999) 122(Pt 3):473–81. 10.1093/brain/122.3.473 10094256

[67] Noble F Kornberg AJ Elder JE Delatycki MB. Retrospective analysis of patients attending a neurofibromatosis type 1 clinic. J Paediatr Child Health. (2007) 43(1–2):55–9. 10.1111/j.1440-1754.2007.01003.x 17207057

[68] Mansouri A Ghadakzadeh S Maqbool T Barnett C Au K Kongkham P Neurofibromatosis clinic: a report on patient demographics and evaluation of the clinic. Can J Neurol Sci. (2017) 44(5):577–88. 10.1017/cjn.2016.326 27821212

